# Development of a wrist and hand stretching device for managing spasticity in stroke patients: a pilot study

**DOI:** 10.3389/fneur.2025.1646697

**Published:** 2025-09-19

**Authors:** Phillip Siwoo Kim, Justin Kim, Philbert Chen, Min Cheol Chang

**Affiliations:** ^1^J&P Robotics, Fullerton, CA, United States; ^2^Carle Illinois College of Medicine, University of Illinois Urbana-Champaign, Urbana, IL, United States; ^3^Department of Rehabilitation Medicine, College of Medicine, Yeungnam University, Daegu, Republic of Korea

**Keywords:** spasticity, stretching therapy, stroke, device, survey

## Abstract

**Objectives:**

We developed a straightforward stretching device for the wrist and hand. To assess the device’s effectiveness in managing spasticity among chronic stroke patients.

**Methods:**

The device, primarily constructed from plastic, comprises a forearm support module, a wrist module, and a finger module. Twenty stroke patients used the device four times daily, 7 days a week, for 1 month. Spasticity severity was measured using the Modified Ashworth Scale (MAS) for the wrist, thumb, and index fingers. A questionnaire evaluated the device’s feasibility and areas for improvement.

**Results:**

Before treatment, the mean MAS scores for the wrist, thumb, and index finger flexors were 1.50 ± 0.36, 1.52 ± 0.34, and 1.50 ± 0.30, respectively, compared with 1.25 ± 0.26, 1.27 ± 0.30, and 1.32 ± 0.33 post-intervention. Patients and occupational therapists expressed satisfaction with the device, citing its ease of use, effectiveness in stretching the wrist and fingers, and overall ease of manipulation. Half of the patients reported that all fingers were easily extended. The rigid plastic finger module was subsequently replaced with an inflatable, flexible rubber ball, providing a more comfortable contour for the stretched fingers, which increased user satisfaction.

**Conclusion:**

The stretching device effectively reduced spasticity in the wrist and hand, and the upgraded device enhanced patient satisfaction.

## 1 Introduction

Spasticity is a form of hypertonus characterized by increased muscle tension in response to stimuli, which intensifies with the velocity of joint movement (1, 2). It is a common sequela of central nervous system disorders, including stroke, traumatic brain injury, spinal cord injury, multiple sclerosis, and cerebral palsy. After a stroke, approximately 65% of patients experience spasticity (3). This condition can lead to muscle tightness and joint stiffness in the affected extremity, resulting in functional disability (4, 5). Therefore, effective management of spasticity is crucial for stroke patients.

Several methods are currently employed to manage spasticity in stroke patients, including oral medications, botulinum toxin or alcohol injections, bracing, serial casting, and stretching exercises (6–10). Among these therapeutic options, stretching exercises, which involve moving joints through their full range of motion via an external force, are one of the most fundamental approaches (11, 12). Stretching exercises alleviate spasticity in stroke patients through several proposed mechanisms (13–15). Prolonged passive stretching reduces stretch reflex excitability primarily via modulation of Ia afferent input to *α*-motor neurons and decreased *γ*-motor drive, leading to reduced sensitivity of muscle spindle (13, 15). Additionally, Golgi tendon organs contribute through autogenic inhibition, further suppressing hyperexcitable motor neuron pools. This, in turn, reduces muscle spindle hyperactivity and promotes viscoelastic changes in muscle-tendon units, thereby increasing joint range of motion (ROM) and reducing resistance to movement. Moreover, repetitive stretching can induce neuroplastic adaptations in supraspinal structures, improving motor control and reducing involuntary muscle contractions.

However, stretching exercises are typically performed manually, requiring a therapist to administer repetitive exercises regularly (11, 16). This manual approach is time-consuming, and outcomes can vary depending on the therapist’s experience. To address these limitations, various stretching devices have been developed, demonstrating positive effects in reducing spasticity (17–20). However, most devices are designed for therapist-assisted use, require considerable time and labor, and are unsuitable for independent operation by patients. If patients could independently wear and use these stretching devices without the assistance of therapists, or with minimal help, it would save therapists’ time and enhance the effectiveness of spasticity management.

We developed a wrist and hand stretching device designed for patients to use conveniently. The device consists of three primary modules: a forearm support module, a wrist module, and a finger module. The forearm support module stabilizes the forearm in a neutral position, reducing strain on the wrist and hand during stretching. The wrist module features a rotational axis that enables controlled wrist extension and can be locked at the desired angle for sustained stretching. The finger module is ergonomically designed to gradually separate the thumb from the other fingers, enabling targeted stretching of the finger flexors. All components are primarily fabricated from lightweight plastic using 3D printing technology, ensuring portability and ease of use without electronic or motorized systems.

We evaluated its effectiveness and assessed feasibility through patient feedback, leading to subsequent device upgrades based on this feedback.

## 2 Methods

### 2.1 Subjects

We prospectively recruited 20 consecutive stroke patients (M:F = 9:11, age = 68.7 ± 4.4, cerebral infarct:cerebral hemorrhage = 7:13, right hemiplegia:left hemiplegia = 11:9, time between onset and start of clinical trial = 13.4 ± 1.1; Fugl-Meyer Upper Extremity = 34.8 ± 7.5; Barthel Index = 72.4 ± 10.2) based on the following inclusion criteria: (1) ≥ 12 months after stroke onset; (2) hemiparesis or hemiplegia due to stroke; (3) sufficient cognitive ability to understand the clinical trial process and respond to our questionnaire (Mini-Mental State Examination score of <25); (4) spasticity in the wrist flexor, thumb flexor, and index finger flexor with a Modified Ashworth Scale (MAS) score between 1 and 2; (5) no history of musculoskeletal disease (e.g., arthritis, musculotendinous injury, or bone fracture) or peripheral nerve injury in the affected upper extremity; (6) no invasive procedure for spasticity management (injection of botulinum toxin, alcohol, or phenol) within 6 months prior to the initiation of this study. The Ethics Committee of the Ministry of Health and Welfare approved this protocol (P01-202410-01-012), and all patients provided written informed consent before participating in the study.

### 2.2 Stretching device and intervention

**Figure 1:**
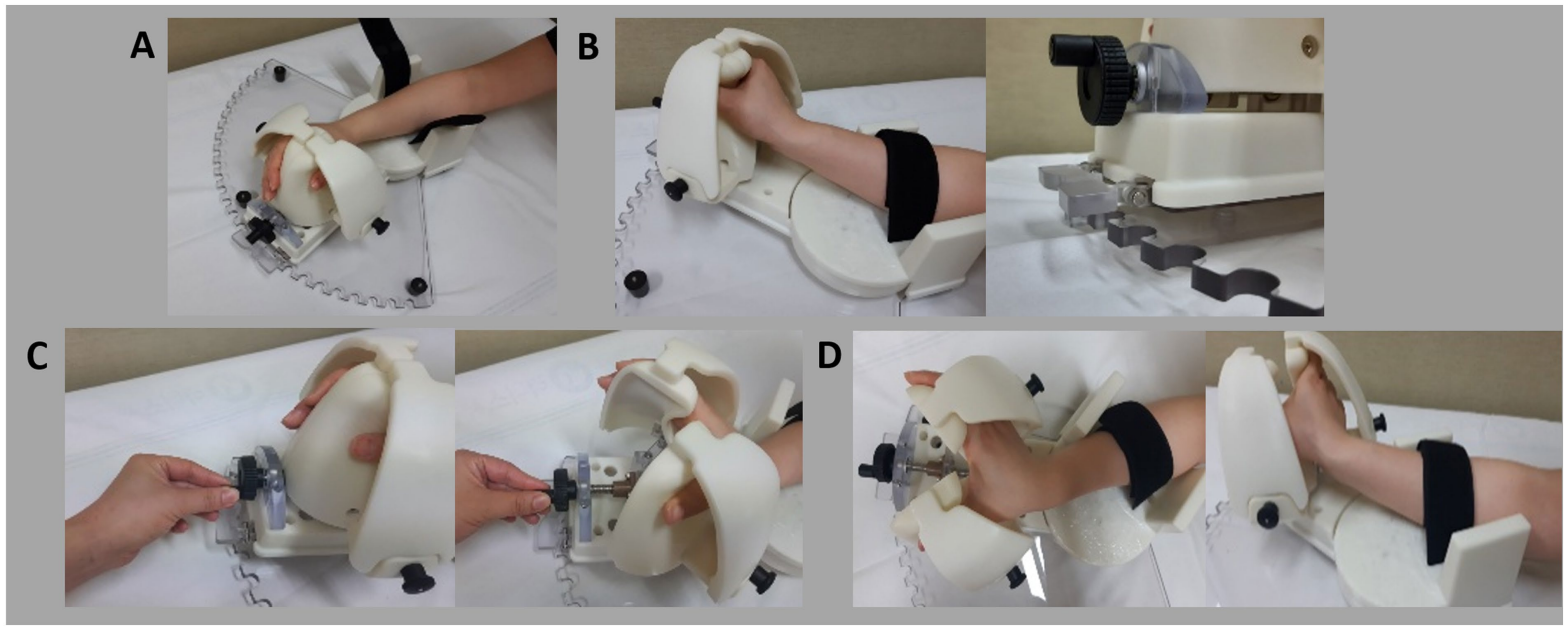
Illustration of device usage. **(A)** Initial setup: Place the patient’s wrist and hand into the device and secure the forearm with Velcro tape. **(B)** Stretch the wrist by rotating the wrist module and locking it in place. **(C)** Stretch the fingers by rotating the wheel. **(D)** Final target position: Fully extended wrist and fingers.

The device comprises a forearm support module, a wrist module, and a finger module. Most components were fabricated from plastic using 3D printing techniques (Figure 1). The device lacks electronic controls or a motorized system, ensuring ease of use and safety for users. The primary function of the device is to stretch and extend the spastic wrist and fingers, allowing individuals to operate it independently without assistance. The forearm support module stabilizes the patient’s forearm during stretching (Figure 1), providing a base upon which the wrist module can rotate to achieve dorsal wrist extension. A rotational axis between the hand and forearm modules allows for easy wrist stretching, and the wrist can be securely fixed at any point using a locking system. The finger module, designed ergonomically for spastic hands and fingers, comfortably and securely holds the patient’s fingers. It stretches the fingers by widening the gap between the thumb and the other fingers, controlled by rotating a wheel. The module can also be fixed at any desired position. The stretching angle for the wrist module was typically set between 45° and 70° of extension. The mechanical design of the device allows a maximum wrist flexion of 90° and a maximum extension of 90°, providing a built-in ROM limit to prevent overstretching. Participants used the device independently, four times daily, 7 days a week, for 1 month. Each stretching session lasted 15 min. The end-point ROM for both the wrist and fingers was determined individually, limited either by patients’ pain tolerance or by firm end-range resistance. Patients’ subjective feedback was used to determine the optimal endpoint, ensuring sufficient stretching intensity to engage target muscles without causing discomfort. Adherence was monitored using daily log sheets completed by patients (or their caregivers, if assistance was required), which recorded session completion. The research team reviewed the logs weekly and conducted brief phone follow-ups to confirm compliance.

### 2.3 Assessment of spasticity

The degree of spasticity was evaluated using the MAS for the wrist flexor, thumb flexor, and index finger flexor (20). All MAS assessments were performed by a single physician with 20 years of experience in stroke rehabilitation to minimize inter-rater variability. Because this was a single-arm pre-post study without random allocation, blinding of the assessor was not applicable. We acknowledge that the assessor was aware of the intervention, which might introduce a potential source of bias. MAS assessments were conducted immediately before the first therapeutic session with the device (pre-treatment) and again 1 day after the final session (post-treatment). To avoid potential confounding from transient changes immediately following stretching, post-treatment MAS evaluations were performed 1 day after the final intervention rather than immediately after a session. The MAS scores were defined as follows: 1—slight increase in muscle tone, indicated by a catch and release or minimal resistance at the end of the ROM during flexion or extension; 1 + —slight increase in muscle tone, indicated by a catch followed by minimal resistance throughout less than half of the ROM; 2—more marked increase in muscle tone through most of the ROM, although the affected part(s) could still be moved easily. For statistical analysis, scores of 1, 1+, and 2 were assigned values of 1, 1.5, and 2, respectively.

### 2.4 User feasibility assessment

A questionnaire was administered to the participants to identify the device’s potential shortcomings and assess its convenience. The questionnaire included the following items:

- Donning and doffing
  - Is it easy to don and doff?
  - How long does it take to don and doff?
  - Does the device provide optimal positioning for the wrist and fingers?
  - How many helpers are required to don and doff?
- Wrist extension
  - Is the wrist module easy to operate?
  - Can the wrist be stretched to the desired angle?
  - Can the extended posture and angle be easily maintained?
- Finger extension
  - Is the finger module easy to operate?
  - Can the fingers be stretched to the desired angle?
  - Can the extended posture and angle be easily maintained?
  - Are all the fingers equally extended and stretched?
- General points
  - Is the device too heavy to carry?
  - Does the baseplate provide firm support?
  - Are there any inconveniences during use?
  - Would you be willing to use the device daily to manage hand spasticity?

### 2.5 Statistical analysis

Data were analyzed using SPSS Statistics for Windows, version 27.0 (IBM Corp., Armonk, NY, USA). To verify normal data distributions, Kolmogorov–Smirnov tests were performed prior to each analysis. As the data were not normally distributed, we compared pre-treatment and post-treatment MAS scores using the Wilcoxon signed-rank test. Statistical significance was set at *p* < 0.05.

## 3 Results

The mean MAS scores at pre-treatment were 1.50 ± 0.36 for the wrist flexor, 1.52 ± 0.34 for the thumb flexor, and 1.50 ± 0.30 for the index finger flexor. After treatment, the mean MAS scores were significantly reduced to 1.25 ± 0.26, 1.27 ± 0.30, and 1.32 ± 0.33, respectively. Statistical analysis confirmed that the reductions in MAS scores for the wrist flexor, thumb flexor, and index finger flexor were significant when compared to pre-treatment values (wrist flexor, *p* = 0.002, *Z* = −3.162; thumb flexor, *p* = 0.002, *Z* = −3.162; index finger flexor, *p* = 0.020, *Z* = −2.333).

**Figure 2:**
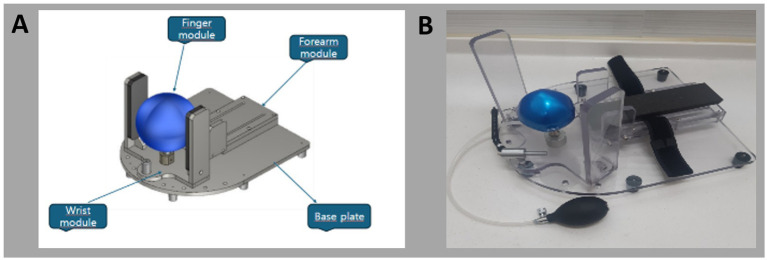
**(A)** 2D schematics and **(B)** photograph of the newly developed device.

**Figure 3:**
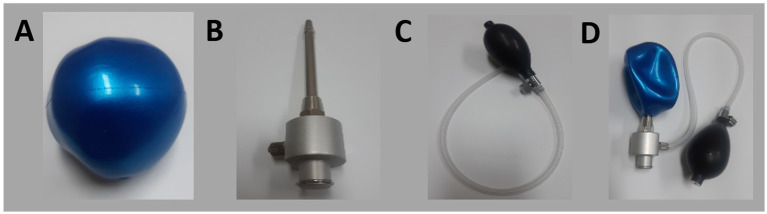
Components of the newly developed device: **(A)** inflatable rubber ball, **(B)** upright bar, **(C)** air pump, **(D)** fully assembled device.

Overall, patient feedback was positive (Table 1). However, when assessing finger extension, only 10 patients (50%) reported that all fingers were easily extended, suggesting that the finger module required improvement to ensure equal extension of all fingers. Based on this feedback, we focused on enhancing the finger module by incorporating a ballooning ball mechanism (Figure 2). This design features a flexible and resilient rubber ball, a metal upright pole, and an air nozzle connected to a manually operated cuff derived from a sphygmomanometer (Figure 3).

**Table 1:** User feasibility test results.

Donning and doffing			
Easy to don and doff	Easy (18)	Moderate (2)	Difficult (0)
Duration (minutes)	<3 (15)	3 ~ 5 (4)	>5 (1)
Optimal positioning	Yes (18)	Moderate (2)	No (0)
How many helpers?	0 (18)	1 (2)	2 (0)
Wrist extension			
Easy to operate?	Easy (20)	Moderate (0)	Difficult (0)
Stretch to wanted angle?	Easy (19)	Moderate (1)	Difficult (0)
Sustain the extended posture?	Easy (20)	Moderate (0)	Difficult (0)
Finger extension			
Easy to operate?	Easy (20)	Moderate (0)	Difficult (0)
Stretch to wanted angle?	Easy (20)	Moderate (0)	Difficult (0)
Sustain the extended posture?	Easy (20)	Moderate (0)	Difficult (0)
All the fingers extended equally?	Easy (10)	Moderate (5)	Difficult (5)
General point			
Too heavy?	No (20)	A bit (0)	Heavy (0)
Firm support?	Yes (20)	A bit (0)	No (0)
Any trouble?	No (18)	A bit (2)	Yes (0)
Willing to use?	Yes (20)	A bit (0)	No (0)

The numbers in parentheses indicate the tallies of patients who responded.

**Figure 4:**
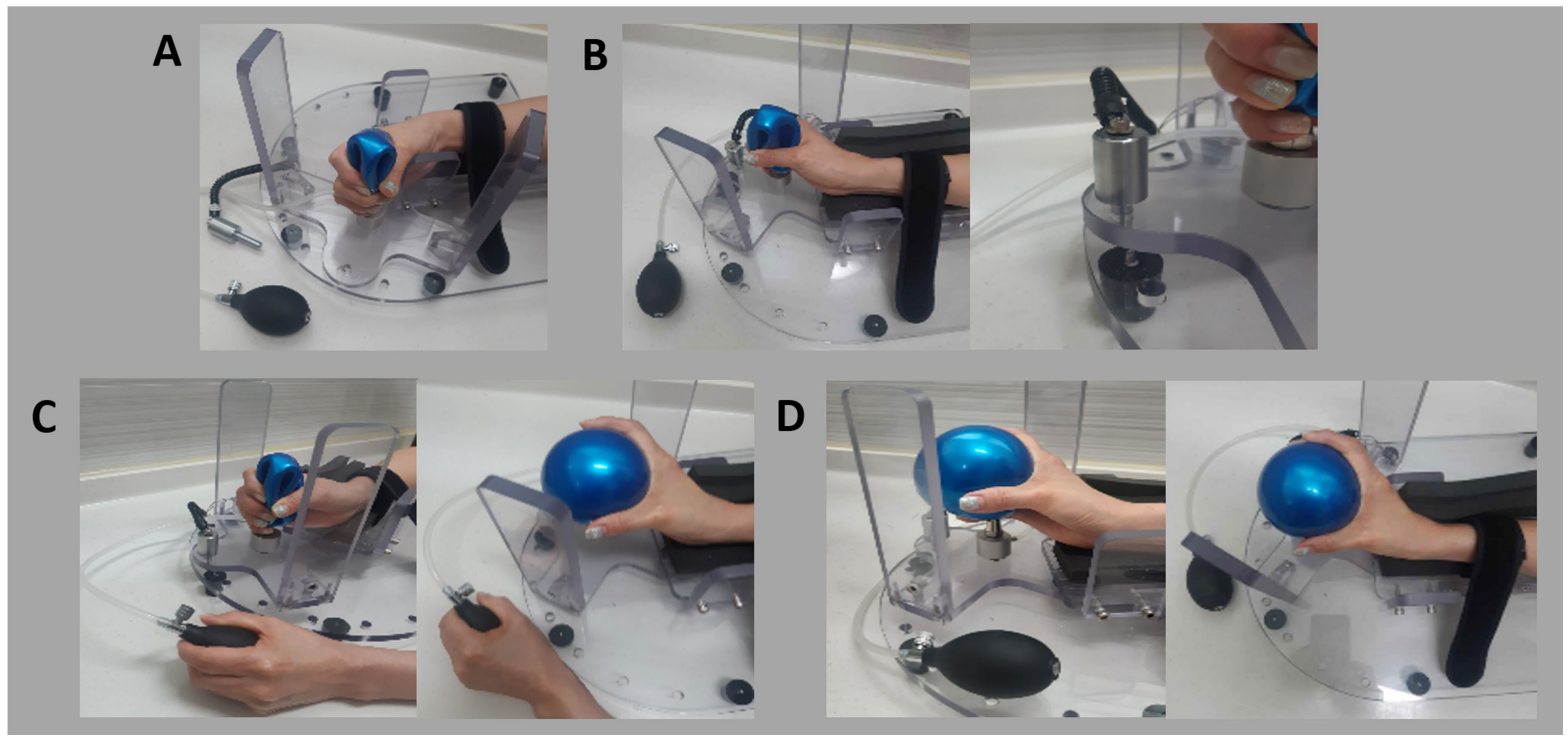
Steps for using the newly developed device: **(A)** Initial setup: Position the patient’s hand in the device and secure the forearm with Velcro tape. **(B)** Stretch the wrist by rotating the wrist module and locking it in place. **(C)** Stretch the fingers by inflating the ball using the cuff. **(D)** Final target position with extended fingers and wrist.

In its resting state, the ball remains deflated, allowing the metal upright pole to support the spastic hand as it fits into the finger module. When the cuff is used to inflate the ball, the expanding ball stretches the spastic hand and fingers (Figure 4). We applied the upgraded device to all 20 patients and reassessed the user feasibility, specifically focusing on finger extension (Table 2). Following the upgrade, all patients reported that all fingers were equally and easily extended. Additionally, all patients indicated that they could easily operate the device, achieve the desired angle of extension, and maintain the extended posture.

**Table 2:** User feasibility test results with the newly developed device.

Finger extension			
Easy to operate?	Easy (20)	Moderate (0)	Difficult (0)
Stretch to wanted angle?	Easy (20)	Moderate (0)	Difficult (0)
Sustain the extended posture?	Easy (20)	Moderate (0)	Difficult (0)
All the fingers extended equally?	Easy (20)	Moderate (0)	Difficult (0)

The numbers in parentheses indicate the tallies of patients who responded.

## 4 Discussion

In this study, we examined the effects of a simple stretching device for the wrist and hand, developed specifically to manage spasticity in chronic stroke patients. After using the device for 1 month, patients exhibited significant reductions in spasticity, as measured by the MAS, in the wrist flexor, thumb flexor, and index finger flexor muscles. Additionally, patient feedback revealed a key limitation of the initial device: not all fingers were easily extended. In response to this feedback, we modified the device by incorporating a ballooning ball mechanism, which effectively allowed for the equal extension of all fingers. All participants reported satisfaction with the modified device.

Previous efforts to develop stretching devices for managing hand spasticity have been made (17–20). However, these devices were limited in their ability to control spasticity in the fingers without addressing wrist spasticity. Additionally, earlier devices often required assistance for donning and operation, limiting their practicality for independent use. In contrast, our device addresses spasticity in both the fingers and wrist and is designed for independent use by the patient. This independence allows patients to use the device as frequently as needed, facilitating more consistent management of spasticity. Feedback from our patient survey further highlighted the device’s ease of use, including its straightforward donning and doffing process, user-friendly module manipulation, and portability.

Furthermore, existing devices such as static splints, continuous passive motion (CPM) devices, and dynamic orthoses show several quantitative shortcomings. Static splints lack angle-control precision, with most providing only fixed extension positions without fine adjustment (14). CPM devices allow repetitive motion but often lack individualized torque or velocity control, resulting in inconsistent therapeutic effects (11). Dynamic orthoses, while more adaptable, still provide limited real-time biofeedback and their angle-control accuracy is typically within 5–10°, insufficient for tailoring to spastic muscles with narrow tolerance ranges. A recent systematic review concluded that many stretching devices fail to achieve reproducible, patient-spastic adjustments and recommended integration of feedback-controlled systems for improved outcomes (10, 11). Compared with these approaches, our device enables individualized adjustment of both wrist and finger joints with mechanical locking at specific extension angles, offering greater reproducibility for independent patient use.

Another critical advantage of our device is its ability to maintain the forearm in a neutral position. Previous devices often positioned the forearm in a prone position, complicating the fitting process and causing discomfort in the spastic upper limb (17–20). When the wrist and fingers are flexed due to spasticity, a prone forearm position hinders proper accommodation of the flexed joints, potentially leading to additional strain on the wrist during stretching. Our device avoids these issues by supporting the forearm in a neutral position, making it easier and more comfortable to fit the spastic limb into the device. Moreover, the device applies stretching forces that are evenly aligned with the wrist and finger joints, preventing undue stress on the joints and ensuring a more effective treatment process.

Our study was conducted without a control group. However, we specifically recruited patients who were at least 12 months post-stroke. Given this timeframe, it is unlikely that the observed reductions in spasticity were due to natural recovery. Previous longitudinal studies have shown that most spontaneous neurological recovery, along with associated reductions in spasticity, occur within the first 3–6 months post-stroke, with minimal further change beyond 12 months (21, 22). Thus, it is unlikely that natural recovery contributed significantly to the improvements observed in our patients. Therefore, although we did not compare the therapeutic outcomes of our stretching device with a control group, our results suggest that the device is effective in managing spasticity in the wrists and hands of chronic stroke patients.

The user survey revealed that patients had some issues with the original finger module, particularly with uneven stretching of the fingers. The rigid plastic module was not well-suited to accommodate spastic fingers, especially at the interphalangeal joints. In response to this feedback, we redesigned the finger module, incorporating an inflatable ball with an upright bar and an air nozzle to serve as a guide pole. This design made it easier for spastic hands to fit into the module. We integrated a puffing device from a sphygmomanometer into the air infusion system, allowing the ballooning ball to provide a flexible and comfortable contour for the fingers, both at rest and during stretching. With the initial device, some severely spastic fingers could escape during stretching, exacerbating the spastic posture. The redesigned module, with its ballooning ball concept, applied fluid pressure evenly across the fingers, ensuring uniform stretching.

The reduction in spasticity observed in this study is clinically meaningful, as it can improve joint mobility and improve function in daily activities, and the absence of adverse events confirms its safety. These effects are likely attributable to biomechanical changes, such as increased muscle-tendon length and improved connective tissue flexibility, as well as neurophysiological mechanisms, including reduced muscle spindle sensitivity and enhanced autogenic inhibition via Golgi tendon organs (23–25).

In conclusion, our stretching device effectively alleviated wrist and hand spasticity in chronic hemiparetic stroke patients, and its feasibility was confirmed through user feedback. Moreover, by incorporating the insights from the user survey, we upgraded the device, leading to enhanced patient satisfaction. However, our study has limitations. It was conducted without a control group, and we did not evaluate the therapeutic outcomes of the modified device. Additionally, we did not monitor serial changes in MAS scores during the 1-month treatment period, nor did we investigate the long-term effects of the treatment. Therefore, further studies are warranted to address these limitations.

## Data Availability

The raw data supporting the conclusions of this article will be made available by the authors, without undue reservation.
